# 663. Two (Plus) Birds, One Stone: The Rapid, Comprehensive, Non-invasive Detection of Co-Pathogens of Critical Importance Using A Plasma-based Microbial Cell-free DNA Next-generation Sequencing Test

**DOI:** 10.1093/ofid/ofab466.860

**Published:** 2021-12-04

**Authors:** Matthew Smollin, Martin S Lindner, Nicholas R Degner, Ricardo Castillo-Galvan, Jose Alexander, Christiaan R de Vries, Ann Macintyre, Bradley Perkins, Asim A Ahmed, Aparna Arun

**Affiliations:** 1 Karius, Inc., Atlanta, Georgia; 2 Karius Inc., San Francisco, California; 3 Karius, Redwood City, California; 4 Karius, Inc, Redwood City, CA

## Abstract

**Background:**

Immunocompromised (IC) patients are at risk for infections by a spectrum of invasive pathogens. The overlap in presentation makes it challenging to differentiate among infectious etiologies and critical co-infections (CI) may remain undiagnosed. Open-ended, comprehensive assessment of infection through microbial cell-free DNA (mcfDNA) next-generation sequencing (NGS) of plasma offers the potential for the rapid identification of multiple co-infecting pathogens of critical importance (CI-POCI) with one test.

**Methods:**

Karius Test^TM^ (KT) results from patients who underwent clinical testing from December 2016 to April 2021 were reviewed for detections of two or more CI-POCI including parasites, fungi (*Pneumocystis jirovecii, Trichosporon sp*, endemic mycoses, *Aspergillus sp*., *Mucorales*, Non-*Aspergillus*/Non-*Mucorales* molds), mycobacteria, *Legionella sp*., *Nocardia sp*. and *Listeria*. KT, developed and validated in Karius’ CLIA certified/CAP accredited lab, detects mcfDNA from plasma. McfDNA is extracted, NGS performed, human sequences removed and remaining sequences aligned to a curated pathogen database of > 1500 organisms. Organisms present above a statistical threshold are reported and quantified. For > 85% of tests the time to result reporting is the next day from sample receipt.

**Results:**

KT detected CI of two or more POCI in 59 samples (75% adults, 25% children). The most common partnering co-pathogens of critical importance were *Aspergillus sp* (38), *Mucorales* (17) and PJP (14); the most common combinations were two or more distinct *Aspergillus sp* (14) followed by an *Aspergillus sp* and a *Mucorales* (12). There were 17 samples with the detection of three or more CI-POCI (29%).

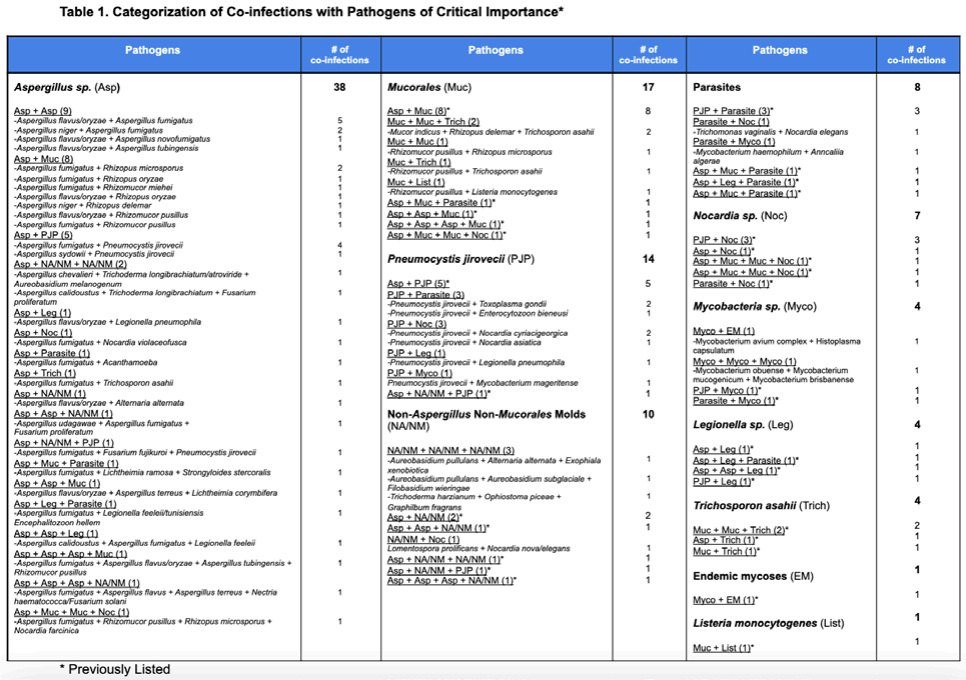

Figure 1. Chord Plot of Co-infections with Pathogens of Critical Importance

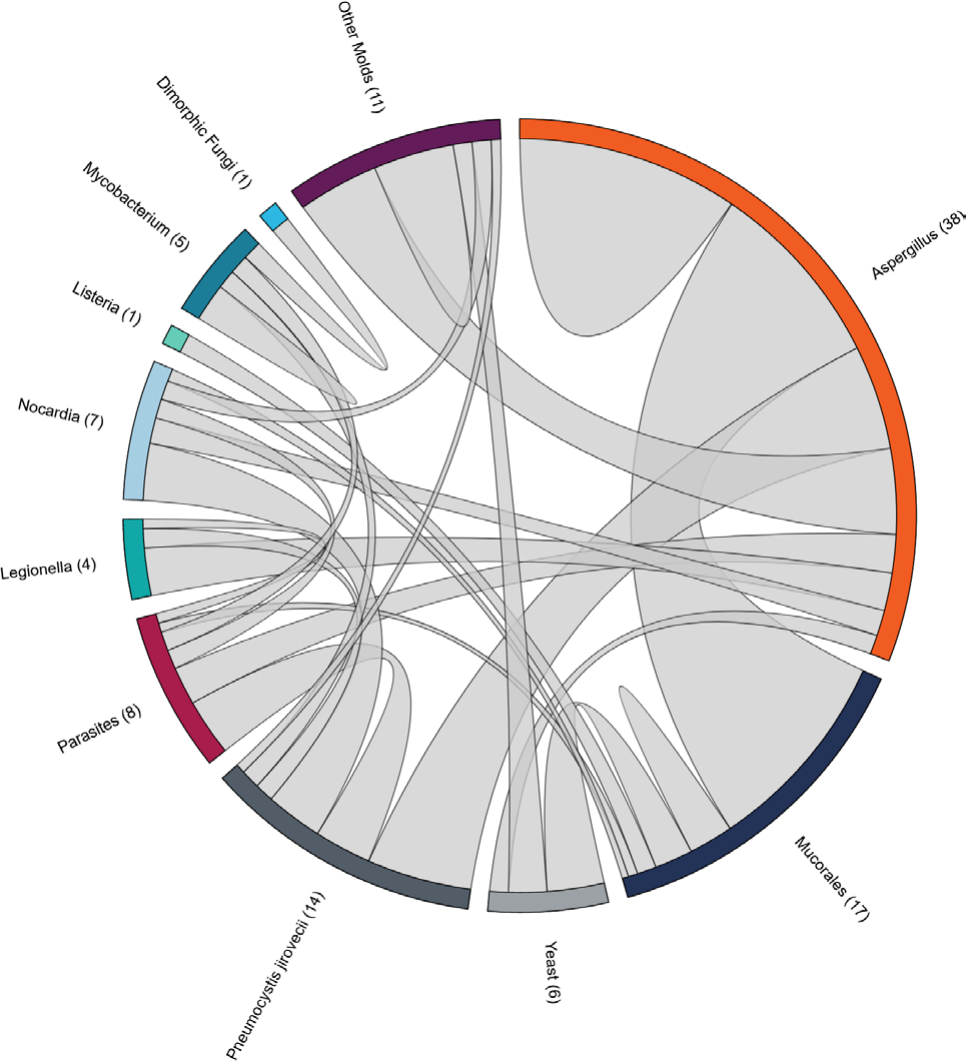

The outer circle sections represent Karius Test detections belonging to different taxonomic groups. The length of each circle section is proportional to the total number of detections of a taxon belonging to that group. The chords connecting a pair of circle sections are proportional to the number of times two taxa from those groups were observed together, weighted by the total number of taxa detected.

**Conclusion:**

Plasma mcfDNA NGS offers a rapid, comprehensive non-invasive means of detecting CI-POCI in IC patients with one test. Although rare, co-infections with POCI can greatly increase mortality. The KT can provide important insights into pathogen-pathogen interactions in complex hosts and help optimize therapy.

**Disclosures:**

**Matthew Smollin, PharmD**, **Karius, Inc.** (Employee) **Martin S. Lindner, PhD**, **Karius, Inc.** (Consultant) **Nicholas R. Degner, MD, MPH, MS**, **Karius Inc.** (Employee, Shareholder) **Ricardo Castillo-Galvan, MD MPH**, **Karius Inc.** (Consultant) **Jose Alexander, MD, D(ABMM), FCCM, CIC, SM, MB(ASCP), BCMAS**, **Karius** (Employee) **Ann Macintyre, DO**, **Karius, Inc.** (Employee) **Bradley Perkins, MD**, **Karius, Inc.** (Employee) **Asim A. Ahmed, MD**, **Karius, Inc.** (Employee) **Aparna Arun, MD**, **Karius** (Employee)

